# Telocytes in the human sinoatrial node

**DOI:** 10.1111/jcmm.13340

**Published:** 2017-11-17

**Authors:** Lubov B. Mitrofanova, Andrey N. Gorshkov, Petr V. Konovalov, Julia S. Krylova

**Affiliations:** ^1^ Department of Pathology Federal Almazov North‐West Medical Research Centre Saint Petersburg Russia; ^2^ Laboratory of Intracellular Signaling and Transport Research Institute of Influenza of the Ministry of Healthcare of the Russian Federation Saint Petersburg Russia; ^3^ Laboratory of Cell Biology Department of Pathology at the FSBI ‘The Ott Research Institute of Obstetrics, Gynaecology and Reproductology’ Saint Petersburg Russia

**Keywords:** telocytes, human sinoatrial node, immunohistochemistry, electron microscopy, confocal laser scanning microscopy

## Abstract

The sinoatrial node (SAN) is composed mostly of pacemaker, transitional and Purkinje‐like cells. Pacemaker cells, especially in the centre of the SAN, are surrounded by dense fibrous tissue and do not have any contact with transitional cells. We hypothesize that the SAN contains telocytes that have contacts with pacemaker cells and contractile myocardium. Immunohistochemistry using antibodies against HCN4 and antibody combinations against CD34 and HCN4 was carried out on 12 specimens. Confocal laser scanning microscopy (CLSM) with two mixtures of primary antibodies, namely CD34/S100 and vimentin/S100, was performed in three cases. In two cases, CLSM was carried out with CD117 antibody. Specimens for electron microscopy and immunocytochemistry with HCN4 immunogold labelling were taken from another three patients. In our study, we found cells with the immunophenotype of telocytes in the SAN. There were twice as many of these cells in the centre of the SAN as in the periphery (20.3 ± 4.8 *versus* 10.8 ± 4.4 per high‐power field). They had close contact with pacemaker cells and contractile cardiomyocytes and expressed HCN4. The ultrastructural characteristics of these cells are identical to those of telocytes observed earlier in other organs. Our study provides evidence that telocytes are present in the SAN.

## Introduction

Although the SAN was described by Keith and Flack as far back as at the beginning of the last century, there has been much controversy over its topography and structure because it shows great variability. Little is known about the human SAN, for example, about the sequence of activation, the expression of ionic currents and the heterogeneity of the tissue 1, 2. In most cases, the SAN is in the subepicardial region. It is composed of dense fibrous tissue that consists of collagen and elastic fibres, among which are ‘walled up’ small round pale P cells—pacemaker cells, 5–10 μm in diameter. They contain a few myofibrils, sparse mitochondria, a poorly developed sarcoplasmic reticulum and lysosomes. P cells typically occur in small groups enveloped together by a basement membrane 3. Cell–cell interactions generally occur through touching cell membranes and partly through desmosomes. The SAN also contains transitional T cells, 12–16 μm in diameter and 40–250 μm long, and large Purkinje‐like cells, 20–50 μm in diameter. T cells can have end‐to‐end, end‐to‐side and side‐to‐side contacts. The myofibrils inside them are arranged at various angles. There are more myofibrils in T cells than in P cells. Purkinje‐like cells have fewer myofibrils but more mitochondria than T cells 4. The SAN is composed mostly of P cells and of large Purkinje‐like cells located in the peripheral region 5. P cells, T cells and Purkinje‐like cells are specialized forms of cardiomyocytes within the SAN. In addition to containing those cell types, the SAN contains fibroblasts, macrophages, mastocytes, pericytes, Schwann cells, blood vessels and nerve fibres. Large bundles of nerve fibres and ganglia are abundant in the region of the SAN, especially in the epicardium. The electrical impulse generated by P cells is believed to be transmitted to the working myocardium through T cells 6. In our opinion, it is interesting that pacemaker cells, especially those in the centre of the SAN, are surrounded by a dense fibrous tissue environment and do not have any contact with transitional cells. How the electrical impulse moves through the heart remains unclear.

Telocytes are a unique type of interstitial cells with telopodes, extremely long but thin prolongations, and dilated segments called podoms. The immunophenotype of telocytes is similar to that of interstitial, endothelial, smooth muscle, mast and haematopoietic stem cells and neurons. They coexpress CD117, vimentin, CD34, SMA, S100 and NSE as well as the gap junction protein Connexin43 7. Telocytes have been observed in the uterus, the uterine tubes, the gastrointestinal tract, the mammary glands, the pancreas, the skin, the ventricular and atrial myocardium and other organs. Apparently, they contribute to pacemaker activity 8. Hyperpolarization‐activated cyclic nucleotide‐gated (HCN) channels present in P cells of the SAN have also been found in the telocytes of the murine gastric atrium 9. HCN channels are integral proteins of cation channels in the membranes of heart cells, the central nervous system and photoreceptor cells. They are referred to as ‘pacemaker channels’, because they contribute to rhythmic activity within heart and brain cells.

Thus, there are two questions to answer: (*i*) How does the electrical impulse move from the P cells to the contractile myocardium of the right atrium? (*ii*) Do we know everything about the cellular components of the SAN?

We hypothesize that the SAN contains telocytes that have contacts with pacemaker cells and contractile myocardium.

### Objective

Morphological study of telocytes in the SAN.

## Materials and methods

All the autopsies were performed in the Federal Almazov North‐West Medical Research (NWMR) Centre (Saint Petersburg). The study was performed in accordance with the principles of the Declaration of Helsinki and approved by the local ethics committee at the Federal Almazov NWMR Centre. Macroscopic examination and histological and immunohistochemical study of the SAN were carried out using post‐mortem material from 12 patients who died from cardiovascular, oncologic and haematologic diseases (cases/patients 1–12 in Table 1); CLSM was performed in five of these cases (No 1–5). The specimens for electron microscopy were taken from another three patients (patients 13–15; seven specimens from each patient). No diseases affected of the SAN in these cases/patients have been reported. All the tissue samples were collected as soon as possible, normally 30–60 min. after death. In total, 15 SANs were studied. The control samples of the working myocardium of the anterior wall of the right atrium for immunohistochemical study and CLSM were obtained from three patients (cases 11, 1 and 2 correspondingly).

**Table 1:** Clinical characteristics of patients and the methods used in our study

No	Sex	Age	Disease	Minimum heart rate	Arrhythmia	Pacemaker	Cause of death	Heart weight (g)	SAN measurements, length × width (mm)	Methods
1	Male	65	DCM	67	Type I, 2nd Degree AV Block	No	HF	563	1.0 × 0.2	H, IHC, CLSM (P)
2	Male	68	CAD	47	No	No	AMI	463	1.2 × 0.2	H, IHC, CLSM (P)
3	Female	28	LVNC	80	Type I, 2nd‐Degree AV Block; VE	CRT	PE	587	1.2 × 0.2	H, IHC, CLSM (P)
4	Female	68	Uterine cancer	65	No	No	Cancer intoxication	386	1.1 × 0.3	H, IHC, CLSM (F)
5	Female	39	Leukaemia	65	No	No	Pneumonia	360	1.0 × 0.4	H, IHC, CLSM (F)
6	Male	41	Aortic valve disease	65	LBBB	bCRT	HF	764	0.8 × 0.5	H, IHC
7	Male	55	DCM	50	No	No	HF	678	0.8 × 0.1	H, IHC, CLSM (P)
8	Female	19	Leukaemia	99	No	No	Pneumonia	320	1.0 × 0.4	H, IHC
9	Male	63	Aortic valve disease	65	No	No	HF	704	0.7 × 0.3	H, IHC
10	Female	48	Uterine cancer	100	No	No	Cancer intoxication	356	1.5 × 0.5	H, IHC
11	Male	69	CAD	70	RBBB	No	AMI	438	1.2 × 0.3	H, IHC
12	Female	72	CAD	86	Paroxysmal AF	No	AMI	479	1.0 × 0.7	H, IHC
13	Male	65	CAD	64	Paroxysmal AF	No	AMI	478	1.0 × 0.2	H, EM
14	Male	46	Leukaemia	82	No	No	Pneumonia	398	1.2 × 0.5	H, EM
15	Male	49	Leukaemia	58	No	No	Pneumonia	378	1.0 × 0.3	H, EM

SAN, sinoatrial node; H, histology; IHC, immunohistochemistry; CLSM, confocal laser scanning microscopy; P, paraffin‐embedded sections; F, frozen sections; EM, electron microscopy; DCM, dilated cardiomyopathy; AMI, acute myocardial infarction; PE, pulmonary embolism; CAD, coronary artery disease; LVNC, left ventricular non‐compaction; HF, heart failure; VE, ventricular extrasystole; RBBB, right bundle branch block; LBBB, left bundle branch block; AF, atrial fibrillation; bCRT, biventricular cardiac resynchronization therapy; AV block, atrioventricular block.

### Macroscopic examination of the SAN

**Figure 1:**
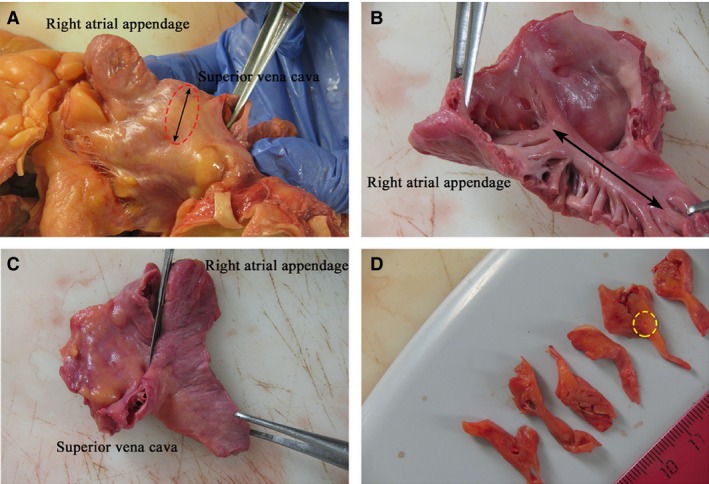
Patient 2 (Table 1). (**A**) The fragment of the anterior wall of the right atrium with the atrial appendage and a part of the superior vena cava. The line with arrowheads at both ends indicates the sulcus terminalis. The sinoatrial node (SAN) area is shown with a circle. (**B**) The fragment of the anterior wall of the right atrium with the atrial appendage and the superior vena cava as seen from the right atrial endocardium. The line with arrowheads at both ends indicates the crista terminalis.(**C**) The serial sections were made parallel to the scalpel in the figure. (**D**) Serial sections of the anterior wall of the right atrium with the SAN. The SAN area is shown with a circle.

To study the SAN, we dissected a fragment of the anterior wall of the right atrium with the atrial appendage, with a part of the superior vena cava approximately 1 cm high and the crista terminalis, in the middle of which was the sulcus terminalis of the right atrium (Fig. 1). The dissection was performed according to the recommendations given by Perde F. V. *et al*. 10. Parallel serial sections were cut transversely to the sulcus terminalis using a scalpel, from the atrial appendage to the superior vena cava, at 3‐mm intervals.

#### Light microscopy

The sections were fixed in 10% neutral‐buffered formalin and embedded in a paraffin block. All the sections were stained with haematoxylin–eosin, van Gieson and Masson's trichrome. The SAN cells were studied morphometrically with routine light microscopy. The specimens were examined with computer‐assisted morphometric analysis using the Leica LAS Image Analysis System (Leica QWin Plus v3, Leica Microsystems IS, Cambridge, UK).

Histological examination of the SAN was carried out using a number of consecutive transverse sections, normally 6–8, made in parallel at fixed intervals (3 mm). The maximum width and length of the SAN on the histological sections were measured in each case. Next, the average size of the SAN was calculated. The cells of the cardiac conduction system were analysed morphometrically, and their diameters were measured at the widest point.

#### Immunohistochemistry

Immunohistochemistry (IHC) using antibodies against HCN4 was carried out in 12 specimens. Double IHC staining using the two‐antibody combination CD34/HCN4 was performed in all the cases.

Paraffin‐embedded sections were first deparaffinized with xylene and rehydrated in a graded ethanol series. To inactivate endogenous peroxidase, the sections were treated with 3% hydrogen peroxide for 5 min. at room temperature and then washed in distilled water. Antigen retrieval was performed with Tris–EDTA buffer (pH 9.0) at 95–98°C for 35 min. Then, the sections were cooled to room temperature. Subsequently, they were washed twice in Tris‐buffered saline with TWEEN 20 (TBST) for 5 min. per wash. Primary antibody incubation was carried out in a container with wet filter paper at room temperature for 30 min. Rabbit polyclonal HCN4 antibody (Alomone Labs, Jerusalem, Israel) at a dilution of 1:100 was used for IHC staining. After staining was complete, the sections were washed twice in TBST. The specimens were incubated with the EnVision Detection Systems Rabbit/Mouse Kit (Dako, Glostrup, Denmark) at room temperature for 30 min. Then, the sections were washed twice in TBST for 5 min. per wash. After being washed in distilled water, the sections were counterstained with haematoxylin for 2 min., dehydrated and then mounted onto the slides using permanent mounting medium (Polystyrol; BioMount, Milan, Italy).

For double‐staining immunohistochemistry, we used mouse monoclonal CD34 antibody at a dilution of 1:50 (clone QBEnd‐10; DAKO) and rabbit polyclonal antibody against HCN4 at a dilution of 1:100 (Alomone Labs, Jerusalem, Israel).

The deparaffinized and dehydrated sections were treated with Tris–EDTA (pH 9.0) at 95–98°C in a water bath for 25 min., cooled down at room temperature for 20 min. and then washed in distilled water. Next, the specimens were incubated in hydrogen peroxidase block solution at room temperature for 10 min. After that, the sections were washed twice in TBST for 5 min. per wash. To reduce nonspecific background staining, the tissue specimens were incubated with UltraVBlock for 10 min. at room temperature. The slices were incubated with a cocktail of CD34/HCN4 primary antibodies at room temperature for 30 min. and washed twice in TBST. In the next step, the sections were incubated with MultiVision anti‐rabbit/HRP + antimouse/AP polymer cocktail (Thermo Scientific, Runcorn, Cheshir, UK) at room temperature for 30 min. and washed twice in TBST. The sections were incubated with LVBlue and LVRed working solutions for 10 min. each. CD34 antigens resulted in blue, HCN4 antigens gave red colour, and the double‐stained cells (their coexpression) were coloured in maroon.

The average number of double‐coloured cells, their average length and the average diameter at the widest point were determined per 10 high‐power fields at 400× magnification. We used the image analysis software Image Scope Colour M (FEI Electron Optics B.V., Moscow, Russia).

### Confocal laser scanning microscopy

CLSM with two mixtures of primary antibodies, namely CD34/S100 and vimentin/S100, was performed in three cases (patients 1–3 in Table 1).

The primary antibodies that we used in our study were mouse monoclonal CD34 antibody at a dilution of 1:50 (clone QBEnd‐10; DAKO), rabbit polyclonal S100 antibody at a dilution of 1:800 (DAKO) and mouse monoclonal vimentin antibody at a dilution of 1:800 (clone V9; DAKO).

The deparaffinized and dehydrated sections were 4–10 μm thick. Heat‐induced epitope retrieval (HIER) with 10 mM citrate buffer (pH 6.0) was performed using a water bath. PBS buffer and TWEEN 20 were used as a wash buffer. Then, the sections were incubated for 30 min. in the blocking serum at room temperature. After a wash, the first primary CD34 and vimentin antibodies were applied for 1 hr at room temperature. We used Alexa Fluor 647® goat antimouse (Abcam, Bristol, UK) as the secondary antibody. After additional washing, the sections were incubated with the second primary S100 antibody for 1 hr at room temperature. Then, we used Alexa Fluor 488® goat anti‐rabbit secondary antibodies (Abcam, Bristol, UK). After another washing, the sections were counterstained with DAPI (appliChem, New Haven, Connecticut, USA). Dako mounting medium was used for mounting all the tissue specimens. When the sections were examined microscopically, the first set of antibodies (CD34, vimentin) showed up in red fluorescence, the second set of antibodies (S100) gave green fluorescence, and the double stain resulted in yellow‐orange fluorescence. The nuclei exhibited contrasting blue fluorescence.

In two cases (cases 4 and 5, see Table 1), CLSM was carried out on the frozen sections to eliminate paraffin autoluminescence. We used primary rabbit c‐Kit (CD117) polyclonal antibody at a dilution of 1:100 (Genemed, San‐Francisco, California, USA). The sections were fixed in 4% PFA for 15 min. and then washed in PBS. The sections were permeabilized in PBS containing 0.1% Triton X‐100 for 20 min. Then, the sections were incubated with blocking serum for 30 min. at room temperature. The incubation with primary CD117 antibodies was carried out in a humidified chamber for an hour at room temperature. We used Alexa Fluor 488® goat anti‐rabbit (Abcam, 1:1000) as the secondary antibody. After another washing, the sections were counterstained with DAPI (appliChem). The prepared specimens were mounted on slides in fluorescent mounting medium (Dako) and covered with coverslips.

The Olympus FV1000D confocal laser scanning microscope was used for fluorescent observation of specimens. The intensity and colocalization (fluorescence) of antigen expression were estimated quantitatively in terms of the standard units (SU, the software of Olympus FV1000D). We used Lazer 647 for murine antibodies (the red fluorescence, the intensity of fluorescence signal was 640–670 SU) and Lazer 488 for rabbit antibodies (the green fluorescence, the intensity of fluorescence signal was 600–650 SU). The offset value was 1%. The frozen sections (cases 4 and 5 in Table 1), 60 μm thick, were scanned at 1.5‐μm intervals in the XY, XZ and YZ planes to generate 3D reconstructions.

### Transmission electron microscopy

Transmission electron microscopy (TEM) was performed on the SAN specimens obtained from three patients (cases 13–15 in Table 1).

The tissue samples were taken 30–60 min. after death. Each specimen was cut into small pieces approximately 1–2 mm^3^ and was pre‐fixed with 2.5% glutaraldehyde in 0.1 M phosphate buffer (pH 7.4) for 45 min. at room temperature. These pieces were washed three times with phosphate buffer and post‐fixed in the same buffer solution containing 1% OsO_4_ for 1 hr. Then, the specimens were dehydrated in a series of ethanol solutions of gradually increasing concentrations and embedded in epoxy resin.

Semithin (1–2 μm) sections were cut from the embedded specimens. These sections were stained with toluidine blue and examined by light microscopy. All the blocks containing myocytes that were somewhat different from the cells of the cardiac contraction system (*e.g*. they were larger in size and had another form) were selected for ultrathin sectioning (Leica EM UC7, Germany/Switzerland). The ultrathin sections were collected onto copper grids, stained with uranyl acetate and lead citrate and examined using a JEM 1011 TEM (JEOL, Tokyo, Japan) equipped with a high‐resolution digital camera (Morada, Olympus, Japan).

For electron microscopic immunocytochemistry, the SAN specimens (patient 15) were first fixed in a PBS solution containing 0.2% glutaraldehyde in addition to 4% paraformaldehyde for 1 hr. Then, the specimens were dehydrated using a series of increasing ethanol concentrations and embedded in LR White resin (Sigma‐Aldrich Inc., St.‐Louis, Missouri, USA). LR White resin was polymerized in tightly capped gelatin capsules at +52°C. Ultrathin sections of samples for TEM (50–70 nm) were cut using an ultramicrotome (Leica EM UC7) and collected on nickel grids. To prevent nonspecific binding (NSB), the sections on grids were incubated in PBS containing 1% bovine serum albumin (BSA‐PBS, Sigma‐Aldrich Inc.) at room temperature for 15 min. An indirect immunogold labelling procedure was used for the detection of HCN4 in the ultrathin sections. Rabbit polyclonal HCN4 antibody at a dilution of 1:100 (Alomone Labs, Jerusalem, Israel) was used as the primary antibody, which is the same antibody we used for IHC examination. The grids with the sections were incubated in the solution of the abovementioned antibody in the phosphate buffer (1:100) for 1 hr and thereafter rinsed in PBS–TWEEN 20 (0.05%). The secondary antibody was 10 nm colloidal gold‐conjugated goat anti‐rabbit IgG (Sigma‐Aldrich, UK) diluted 1:100 and incubated for 1 hr. Then, the grids with the sections were stained in aqueous solutions of uranyl acetate followed by lead citrate.

The sections were examined with a transmission electron microscope (JEM‐1011, JEOL Corp.). Electron micrographs were taken with a Morada digital camera (Olympus, Japan).

### Statistics

Statistica software (v10.0, StatSoft Inc., Palo Alto, California, USA) was used for statistical analysis. Comparisons of continuous variables were made using analysis of variance (anova) and Student's *t*‐test. Categorical variables were compared using Fisher's exact test. Significant differences between groups were defined as those at *P* < 0.05.

## Results

### Light microscopy

**Figure 2:**
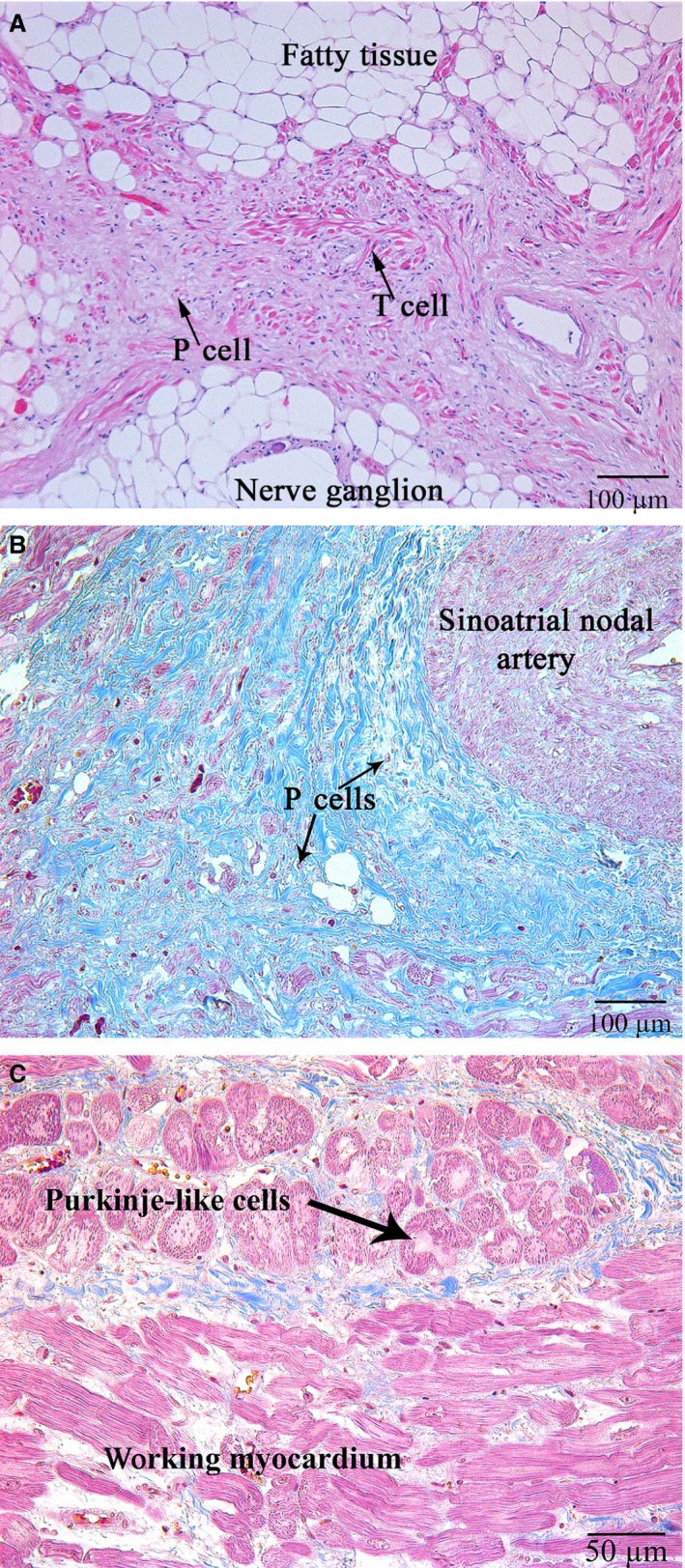
(**A**) Patient 11 (Table 1). The sinoatrial node (SAN). Specialized cells of the cardiac conduction system (thin black arrows); haematoxylin and eosin, ×100. (**B**) Patient 13 (Table 1). Specialized pacemaker cells of the cardiac conduction system are “walled up” in the dense fibrous tissue (thin black arrows); the sinoatrial nodal artery is shown with a star; Masson's trichrome stain, ×100. (**C**) Patient 13 (Table 1). Purkinje‐like cells at the margins of the SAN (shown with a black arrow); haematoxylin and eosin, ×200.

For the cases under study, the average size of the SAN on the histological sections was 1.0 ± 0.2 × 0.3 ± 0.2 cm. The SAN consisted of round P cells with pale cytoplasm, 3.6–8.2 μm in diameter, 4.6 ± 3.1 μm on average, which occurred alone, in pairs or in small groups and were walled up in dense fibrous tissue. They did not have any contact with other special cells of the cardiac conduction system (Fig. 2). The T cells making contact with each other end‐to‐side and side‐to‐side were 6–14 μm in diameter, 11.6 ± 4.3 μm on average. Purkinje‐like cells were located at the margins of the SAN, had pale cytoplasm and were 25–35 μm in diameter, 29.2 ± 10.7 μm on average. The sinoatrial nodal artery passed through the node. In two of 10 cases, it was not in the middle of the node but in the peripheral region. The SAN was surrounded by adipose tissue, nerve fibres and ganglia.

### Immunohistochemical study

**Figure 3:**
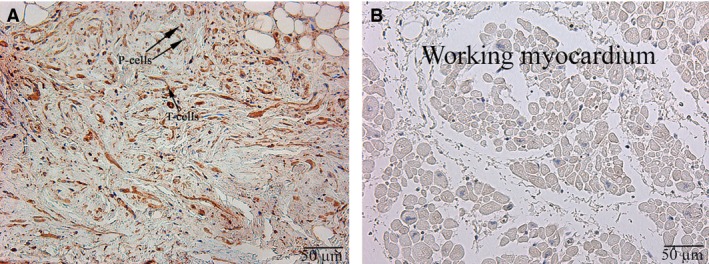
Patient 11 (Table 1). (**A**) The sinoatrial node (SAN). (**B**) The working myocardium of right atrium (control). P cells, pacemaker cells, T cells, transitional cells (indicated with arrows). HCN4; ×200.

**Figure 4:**
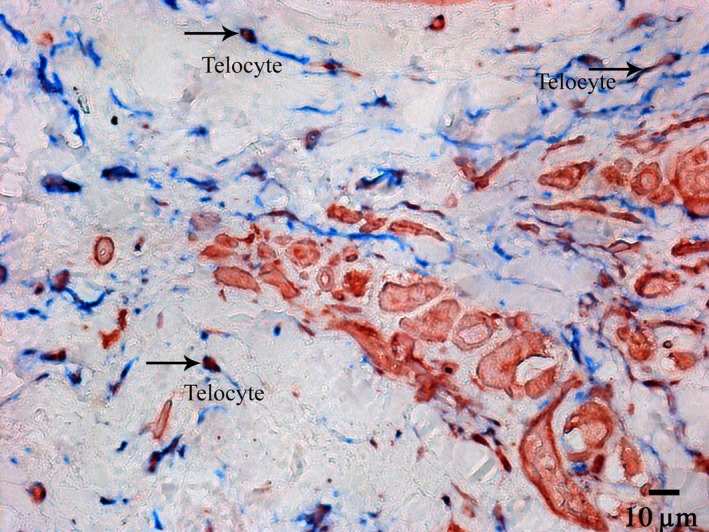
Patient 8 (Table 1). Double staining of HCN4 (red) and CD34 (blue). Cells of telocyte immunophenotype are coloured maroon (indicated with arrows). Cells of the cardiac conduction system are coloured red; ×400.

Immunohistochemistry staining using HCN4 showed that all the specialized cells of the cardiac conduction system expressed this marker (Fig. 3). Double IHC staining revealed coexpression of HCN4 and CD34 in triangular, oval or piriform cells with long and thin processes (the structure typical for telocytes; Fig. 4).

The average quantity of cells of telocyte immunophenotype per high‐power field (400× magnification) was 16.2 ± 4.5. It should be stressed that the cells were irregularly distributed in the SA node. The highest quantity of cells (20.3 ± 4.8) was located in the central part of the node, whereas the average quality of cells at the margins, mainly in adipose tissue, was 10.8 ± 4.4. The average cellular length was 29.2 ± 12.4 μm, and the average diameter at the widest point (of triangular, oval or piriform cells) was 2.6 ± 0.6 μm.

### Confocal laser scanning microscopy

**Figure 5:**
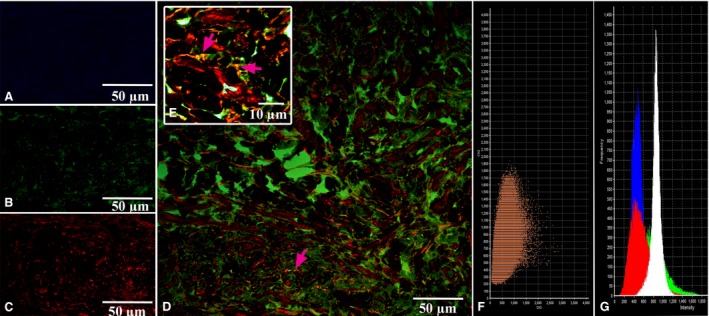
Patient 1 (Table 1). Laser confocal scanning microscopy of the sinoatrial node (SAN). (**A**) Blue fluorescence of cell nuclei (DAPI). (**B**) Green fluorescence of S100. (**C**) Red fluorescence of CD34. (**D, E**) Coexpression of CD34 and S100 on the telocytes is visualized with yellow/orange colour (arrows). A, B, C, D: ×100. E (inserted): ×900. (**F**) The scatterplot for the colocalization of S100 (ch2) and CD34 (ch3). (**G**) The intensity histograms of green (S100), red (CD34), and blue (DAPI) fluorescence and white (differential interference contrast (DIC) image. Figures F and G show the fluorescence intensity and the colocalization of the markers being studied and their quantitative levels.

**Figure 6:**
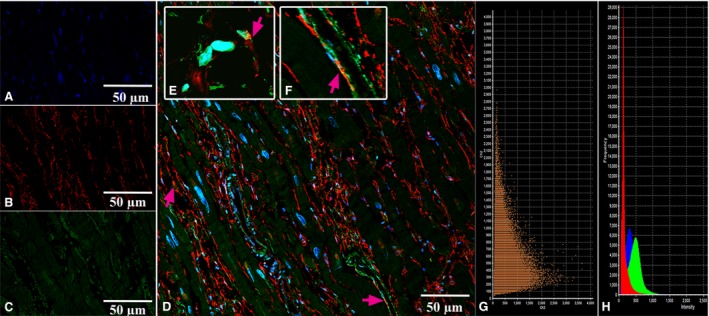
Patient 1 (Table 1). Laser confocal scanning microscopy of the working myocardium of right atrium (control). (**A**) Blue fluorescence of cell nuclei (DAPI). (**B**) Red fluorescence of CD34. (**C**) Green fluorescence of S100. (**D, E, F**) Coexpression of CD34 and S100 on the telocytes is visualized with yellow/orange colour (arrows). A, B, C, D: ×100. E,F (insert): ×900. (**G**) The scatterplot of the colocalization of S100 (ch2) and CD34 (ch3). (**H**) The intensity histograms of green (S100), red (CD34) and blue (DAPI) fluorescence. Figures G and H show the fluorescence intensity and the colocalization of the markers being studied and their quantitative levels.

**Figure 7:**
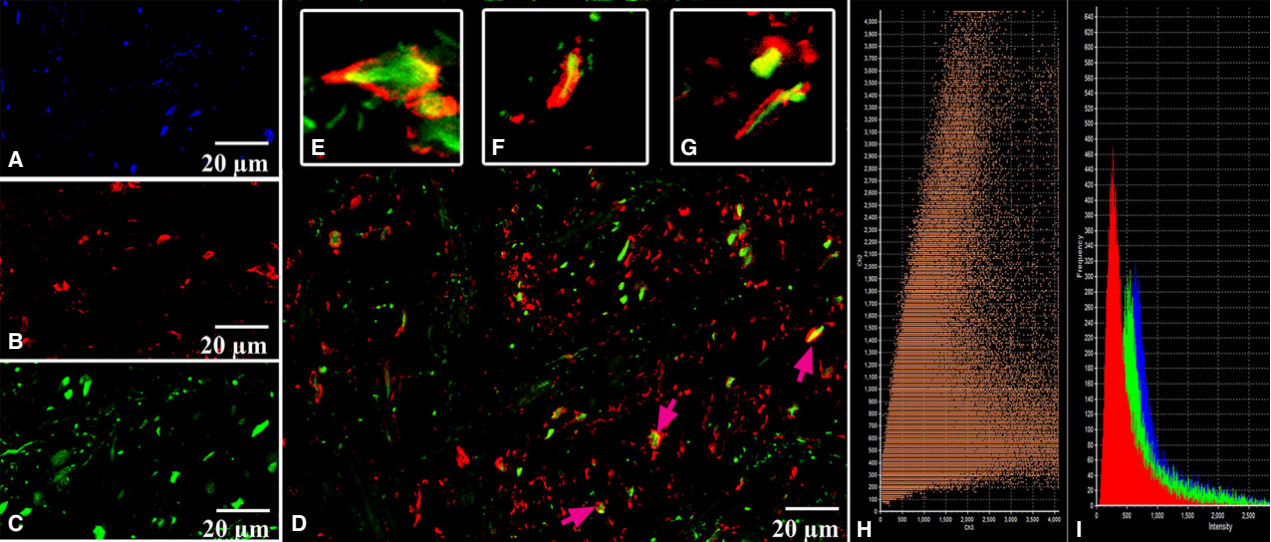
Patient 2 (Table 1). Laser confocal scanning microscopy of the sinoatrial node (SAN). (**A**) Blue fluorescence of cell nuclei (DAPI). (**B**) Red fluorescence of vimentin. (**C**) Green fluorescence of S100. (**D, E, F, G**) Coexpression of vimentin and S100 on the telopodes of telocytes is visualized with yellow/orange colour (arrows). A, B, C, D: ×600. E, F, G: Zoom. (**H**) Scatterplot of colocalization of S100 (ch2) and vimentin (ch3). (**I**) The intensity histograms of green (S100), red (Vimentin) and blue (DAPI) fluorescence. Figures H and I show the fluorescence intensity and the colocalization of the markers being studied and their quantitative levels.

**Figure 8:**
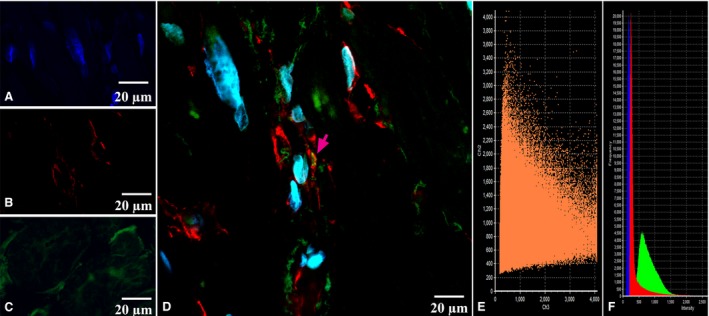
Patient 2 (Table 1). Laser confocal scanning microscopy of the working myocardium of right atrium (control). (**A**) Blue fluorescence of cell nuclei (DAPI). (**B**) Red fluorescence of vimentin. (**C**) Green fluorescence of S100. (**D**) Coexpression of vimentin and S100 on the telopodes of telocytes is visualized with yellow/orange colour (arrows). A, B, C, D: ×600. (**E**) The scatterplot of the colocalization of S100 (ch2) and vimentin (ch3). (**F**) The intensity histograms of green (S100), red (Vimentin) and blue (DAPI) fluorescence. Figures E and F show the fluorescence intensity and the colocalization of the markers being studied and their quantitative levels.

**Figure 9:**
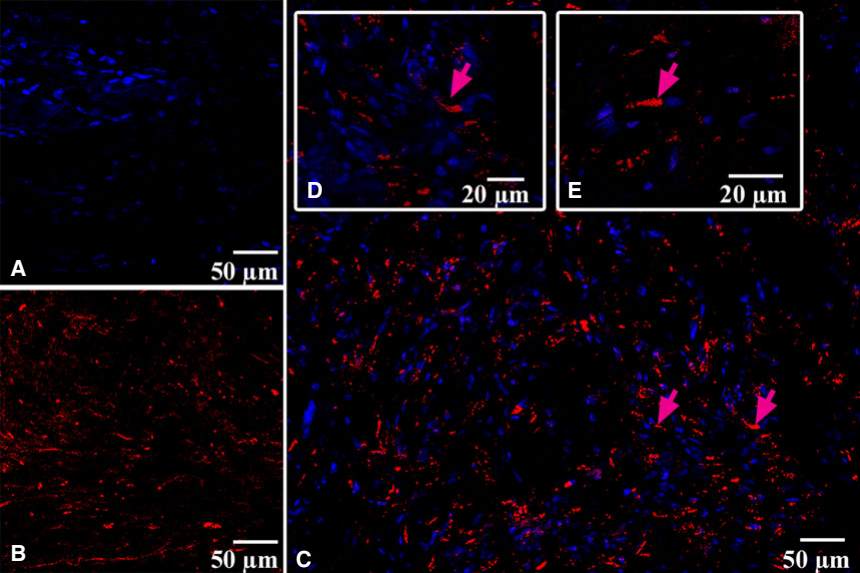
Patient 4 (Table 1). Laser confocal scanning microscopy of the sinoatrial node (SAN). (**A**) Blue fluorescence of cell nuclei (DAPI). (**B**) Red fluorescence of CD117. (**C, D, E**) Expression of CD117 on the telocytes is visualized with red colour (arrows). A, B, C: x200; D: x400; E: ×600.

**Figure 10:**
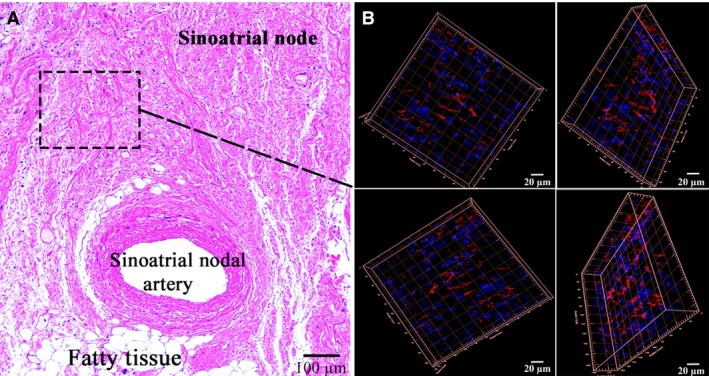
Patient 5 (Table 1). (**A**) The sinoatrial node (SAN); haematoxylin and eosin, ×200. (**B**) Laser confocal scanning microscopy of the SAN with 3D reconstruction (framed). Blue fluorescence of cell nuclei (DAPI). Red fluorescence of CD117; ×200.

CLSM gave us an opportunity to observe the coexpression of CD34 with S100 and of S100 with vimentin in the above‐described cells with some long and thin prolongations corresponding to the telocyte immunophenotype (Figs 5, 6, 7, 8). In the first antibody cocktail, the mean expression (fluorescence) intensity of CD34 was 528 ± 224 (in the working myocardium – 173 ± 122) SU and that of S100 was 728 ± 270 (in the working myocardium – 469 ± 200) SU. In the second antibody cocktail, the mean expression (fluorescence) intensity of vimentin was 518 ± 446 (in the working myocardium – 325 ± 229) SU and that of S100 was 520 ± 456 (in the working myocardium – 430 ± 329) SU. Coexpression of vimentin and S100 was observed as orange fluorescence on the telopodes establishing contacts with nerve fibres (Fig. 7). CD117 expression (fluorescence) occurred in a great number of cells of the SAN, including the piriform cells with long and thin processes (Fig. 9). The 3D reconstruction showed a dense network of long prolongations of telocytes called telopodes (Fig. 10).

### Transmission electron microscopy

**Figure 11:**
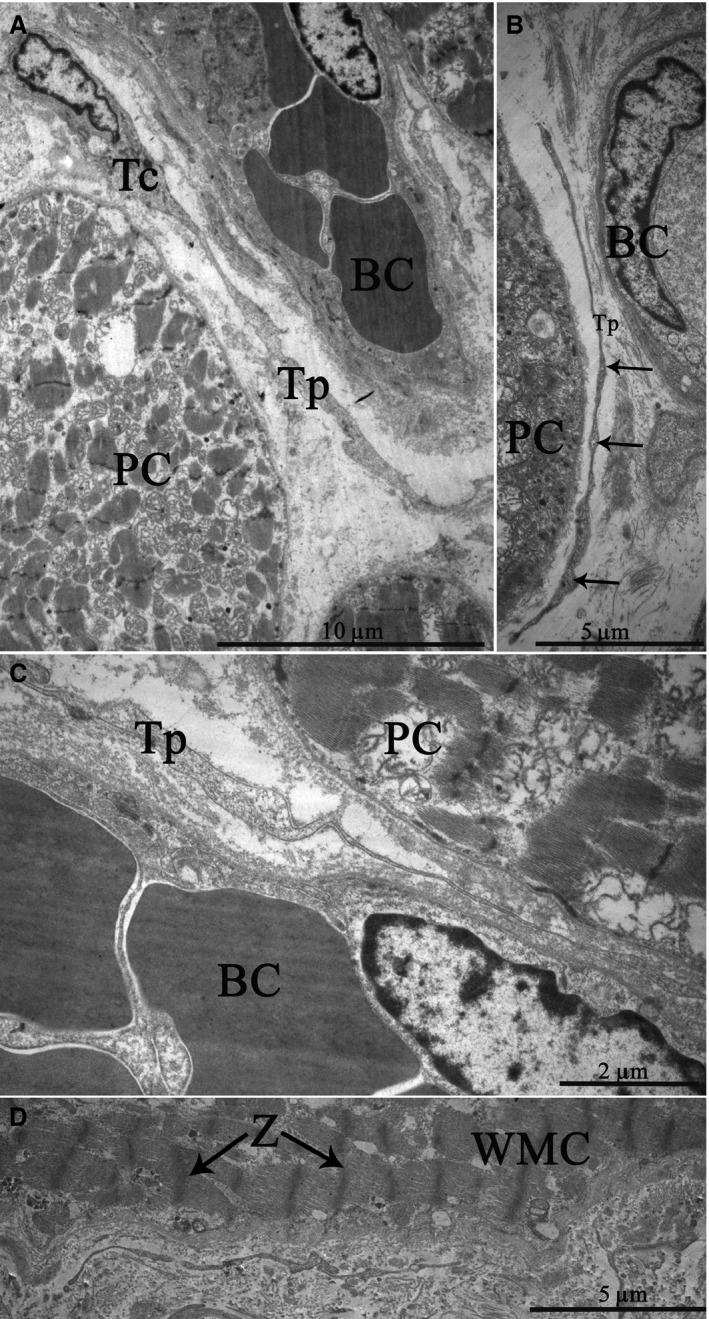
Patient 13 (Table 1). Transmission electron microscopy. The ultrastructure of telocytes in the sinoatrial node (SAN). (**A**) a cell body with a single nucleus and a telopode extending away; (**B**) dilated segments termed “podoms” within a telopode (arrows); (**C**) a telopode located near a blood capillary and a P cell. (**D**) a telopode located close to a working myocardial cell at the SAN periphery. Tc, telocyte, N, nucleus of telocyte, Tp, telopode, BC, blood capillary, PC, P cell, WMC, working myocardial cell, Z, Z‐discs (arrows).

TEM has revealed that the interstitial tissues of the SAN usually contain cells with a relatively small cell body of 6–12 μm and with a single nucleus (Fig. 11A). These cells have long, thin, sometimes tortuous cytoplasmic prolongations, termed telopodes, 0.1–0.3 μm thick and more than 40 μm long. Measuring the real length of telopodes on the ultrathin sections is difficult because telopodes have uneven shapes and could be only partially contained on the section plane. The ultrastructural characteristics of these cells have been identical to those of telocytes observed earlier in other organs and other heart parts 11. A specific characteristic of telopodes is their moniliform aspect, an alternation of long, thin prolongations called ‘podomeres’ and dilated segments called ‘podoms’ that are 0.4–0.8 μm thick (Fig. 11B). In the SAN, telocytes and their telopodes are mainly located near the specialized cells of the cardiac conduction system and close to fine blood vessels (Fig. 11A,B,C). At the periphery of the SA node, telopodes are quite often located close to the working myocardial cells composed of long bundles of myofibrils and well‐ordered Z‐discs (Fig. 11D).

**Figure 12:**
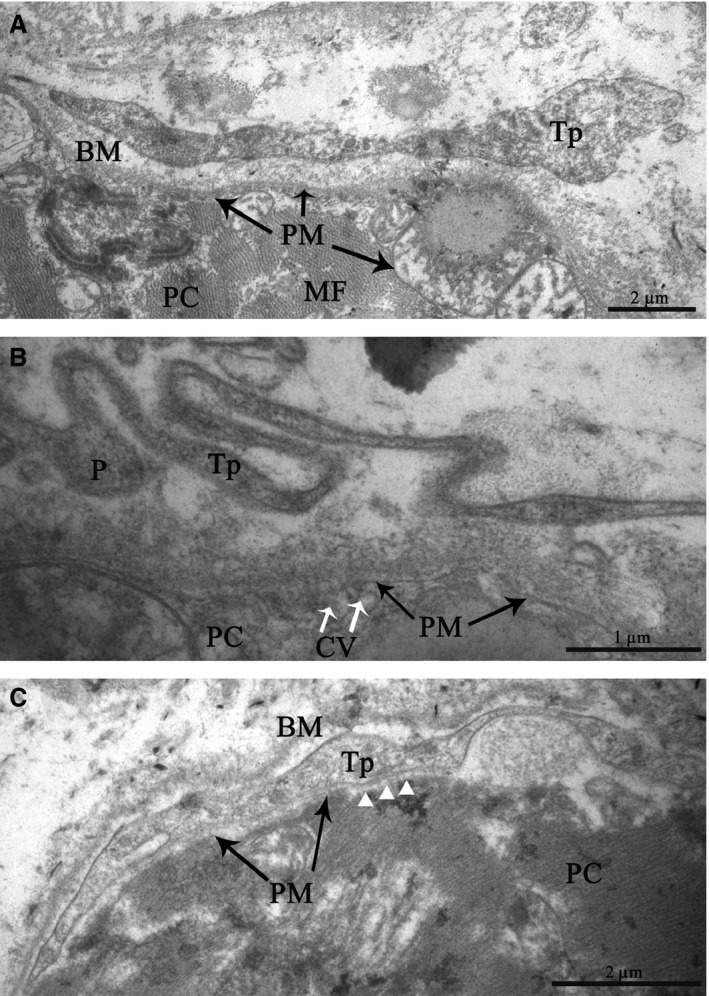
Patient 14 (Table 1). The sinoatrial node (SAN). Transmission electron microscopy. Spatial relationships between telopodes and P cells in details. (**A, B**) Telopodes are in close vicinity to the basement membrane enclosing a P cell. (**C**) The penetration of a telopode through the basement membrane of a P cell. The intercellular junction with electron‐dense structures connecting the membranes of a telopode and a P cell (arrow heads). Tp, telopode, P, podom, BM, basement membrane, PC, P cell, PM, the plasma membrane of P cell, MF, myofilaments, ID, intercalated disc, CV, caveolae

Telopodes are often located near and along the basement membrane enclosing the specialized cardiomyocytes of the cardiac conduction system at a distance of 0.2–0.4 μm from their plasma membrane (Fig. 12A,B). In some cases, we have found that telopodes penetrated the basement membranes of P cells; telopodes and P cells approached each other from up to 20–30 nm away and formed intercellular junctions (Fig. 12C). Electron‐dense rod‐like structures that maintained the structural association of membranes as a part of contacts have been apparent.

**Figure 13:**
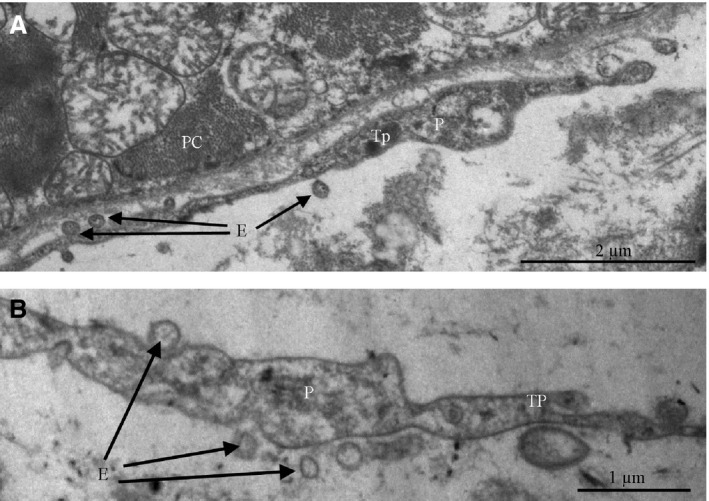
Patient 14 (Table 1). (**A, B**) Extracellular vesicles (exosomes) budding from the plasma membrane of a telocyte into the region of a telopode. Tp, telopode, P, podom, E, exosomes (arrows), PC, P cell.

Telocytes regularly release vesicles, 100–200 nm in diameter, into the extracellular environment. These vesicles have been observed budding from the plasma membrane of a telopode (Fig. 13). Based on the size, they can be classified as exosomes. Exosomes are known to serve as cargo containers to transmit signalling molecules from one cell to another in various organs 12, particularly in the heart 13. Therefore, we suppose that these vesicles are a means of cell‐to‐cell communication between telocytes and surrounding cells.

**Figure 14:**
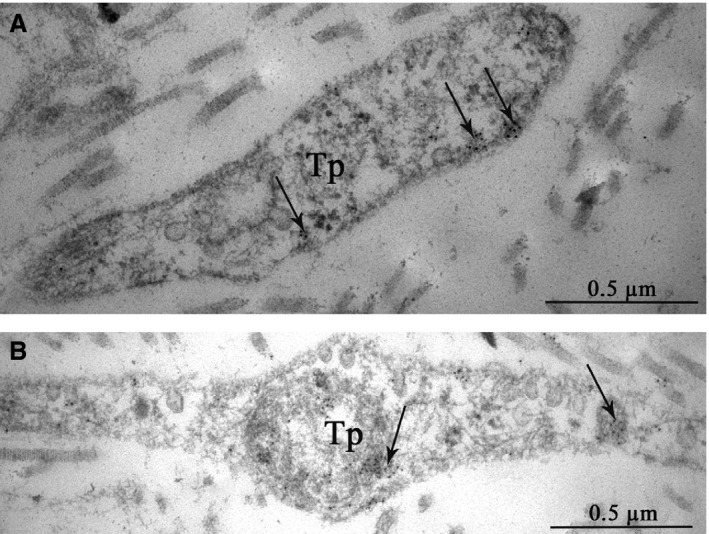
Patient 15 (Table 1). Indirect immunogold labelling of HCN4 in telocytes of the SAN. HCN4 is present in telopodes as small clusters of colloidal gold (**A, B**), predominantly on the plasma membrane (arrows). Tp, telopodes.

HCN4 immunogold labelling on ultrathin sections of the SAN allowed us to detect this protein in telopodes, which is consistent with our IHC findings (see Fig. 4). Indirect immunolabelling revealed HCN4 in the form of small clusters of colloidal gold mainly on the plasma membrane of telopodes (Fig. 14).

## Discussion

In this study, using TEM, IHC and CLSM, we have shown that telocytes are present in the SAN. The telocytes expressed CD117 and the combinations of HCN4 and CD34, CD34 and S100, and S100 and vimentin.

Many researchers have proven the heterogeneity of the SAN in terms of electrophysiological characteristics, such as pacemaker activity, action potential configuration and conduction, densities of ionic currents, expression of gap junction proteins (Cx40, Cx43 and Cx45), autonomic regulation and ageing‐related changes 14, 15, 16. In our study, we have provided results supporting a heterogeneous structure of the SAN based on morphological examination using CLSM and TEM. In the context of electrophysiology, the SAN is a heterogeneous structure that expresses a unique set of cardiac ion channels necessary for the generation and propagation of the action potential 17. Verkerk A.O. *et al*. first showed the role of the funny current in pacemaker activity and in determining heart rate in the human SAN 18. Ion channel genes encoding this current belong to the HCN gene family, which consists of four isoforms. Three of the isoforms occur in the human SAN: HCN1, HCN2 and HCN4. The HCN1 and HCN4 isoforms are believed to be prevalent in the human heart. It is well known that HCN1 shows the fastest activation kinetics and that HCN4 has slow gating kinetics 19.

Boyett M.R. *et al*. studied the heterogeneity of electrical activity throughout the SAN and revealed gradual change from the periphery to the centre: electrical impulses pass faster in the periphery 20. In our study, we have provided evidence for HCN4 expression by telocytes, but it is well known that HCN4 differs from HCN1 and HCN2 by its unusually slow gating kinetics 21. Slow transmission of electrical impulses in the centre of the SAN may be connected with the fact that there are twice as many telocytes there as in the periphery.

In our opinion, Brioschi C. *et al*. 22, writing about HCN4‐positive cells organized in isolated small multicellular aggregates (islets) and bridged by thin cytoplasmic extensions to form a three‐dimensional mesh‐like structure, actually described P cells entwined into a network of telocytes. The same authors asserted that rabbit SAN myocytes with a high level of HCN4 expression were pacemaker cells. Our results have shown that a high intensity (density) of HCN4 expression in the central part of the SAN is caused not only by P cells but also by a great number of telocytes present there.

We have also demonstrated that telocytes are located beside both P cells and working myocardium on the border of the SAN. The myocardium contains a 3D network of telocytes that tightly cover cardiomyocytes and are involved in contacts with all types of cells and structures. The modulating effect of telocytes on myocytes, immunocytes, vascular cells and nerve cells was confirmed 23. In addition to gap junctions, telocytes have point contacts, nanocontacts and planar contacts with immunocytes, nerve cells, endotheliocytes, pericytes, Schwann cells and cardiomyocytes. Telocytes are often found in close proximity to the basement membrane of cardiomyocytes, but the distance between the cell membrane of the telocyte and that of the cardiomyocyte is approximately 150 nm. Nonetheless, there are dot fusions and dot junctions connecting the cellular membranes. It is interesting to note that electron‐dense Z‐band‐like material has been observed in the cardiomyocyte cytoplasm. Telocyte/cardiomyocyte junctions are normally located in the region of intercalated discs and less often located outside this region. We have observed intercellular contacts between pacemaker cells and telocytes as electron‐dense structures (nanocontacts) arranged in parallel with one another and perpendicular to membranes, and we have also observed planar contacts between telopodes and cardiomyocyte membranes. Telocytes penetrated the basement membrane that enveloped two P cells together. We have found vesicles, 100–200 nm in diameter, budding from the plasma membrane of a telocyte in the immediate vicinity of a P cell. Depending on their size, these vesicles can be classified as exosomes and/or microvesicles 24. A similar observation with telocytes in the atrial myocardium was made by Kostin S. and Popescu L.M. 25. While exosomes have been ‘classically’ described as vesicles originating from the endocytic pathway through fusion of multivesicular bodies in the cytoplasm, direct budding from the plasma membrane has also been described 26. It has been convincingly shown that exosomes are able to mediate cell‐to‐cell communication, serving as delivery vehicles for signalling molecules (proteins, microRNAs, *etc*.) from one cell to another (reviewed in 27).

## Conclusion

The findings of our study provide evidence that telocytes are present in the SAN. Further studies are warranted to investigate the electrophysiological characteristics of telocytes and their role in electrical conduction.

## Conflict of interest

The authors confirm that there are no conflicts of interest.
